# Is kinesiophobia a predictor of early functional performance after total hip replacement? A prospective prognostic cohort study

**DOI:** 10.1186/s12891-020-03748-7

**Published:** 2020-11-07

**Authors:** M. Morri, E. Venturini, N. Franchini, R. Ruisi, A. Culcasi, A. Ruggiero, C. Govoni, M. G. Benedetti

**Affiliations:** 1 IRCCS Istituto Ortopedico Rizzoli, Servizio di Assistenza Infermieristico Tecnico e Riabilitativo, Via Pupilli 1, 40136 Bologna, IT Italy; 2 IRCCS Istituto Ortopedico Rizzoli, Servizio di Medicina Fisica e Riabilitativa, Bologna, Italy

**Keywords:** Osteoarthritis, Arthroplasty, Psychology, Recovery of function

## Abstract

**Background:**

Considerable attention has been paid to the role of kinesiophobia with respect to knee prosthesis but it has not yet been studied as a prognostic factor of short-term functional performance following total hip replacement. The main purpose of the present study is to examine the possible predictors of early functional performance of patients undergoing total primary hip arthroplasty, including demographics as age, sex and body mass index, preoperative functional ability, type of anaesthesia, level of haemoglobin, pain and level of kinesiophobia before surgery. Secondly, we want to describe the main characteristics of the population with the highest levels of kinesiophobia.

**Methods:**

A prospective, prognostic cohort study was carried out. Patients undergoing primary hip replacement were recruited consecutively. The main outcome is the early functional performance achieved by patients after surgery and measured using the Iowa Level of Assistance (ILOA) scale on the fifth postoperative day. Preoperative kinesiophobia was measured by the Tampa Scale and the preoperative functional ability by the Western Ontario and McMaster Osteoarthritis Index (WOMAC). The multivariate analysis was performed by the General Linear Model. The analysis of the population with high levels of kinesiophobia was conducted by identifying a cut-off of 40 compared to the Tampa Scale.

**Results:**

Statistical analysis was performed on 269 patients. The average ILOA score recorded was 19.5 (DS 8.3). The levels of kinesiophobia, showed an average score of 35.1 (7.8) and it was not associated with early functional performance. The independent predictive factors include age, sex and body mass index. Kinesiophobia high levels were recorded in 30% of the population and this population had a higher level of pre-operative WOMAC score.

**Conclusions:**

Early functional performance after hip replacement surgery was not correlated with the level of kinesiophobia. Three significant factors that describe a population most at risk of not achieving optimal functional performance are increased age, being female and increase in body mass index. In the preoperative phase, high levels of kinesiophobia were associated with more impaired preoperative functional ability.

**Trial registration:**

Current Controlled Trials NCT02786121, May 2016. Retrospectively registered.

## Background

Total hip replacement generally has a good level of success, usually guaranteeing complete restoration of joint function and elimination of painful symptoms [1, 2]. However, in a percentage of patients, the risk of non-optimal functional recovery remains [3, 4]. The planning of care pathways in today’s orthopedic surgery units continuously require the need to combine the reduction in hospitalization time with early motor skills recovery. The possibility to identify patients at risk by determining the correct outcome predictors is a critical element in this context. Currently, factors that show to have significant roles in hip replacement surgery are age, sex, body mass index (BMI) and pre-operative functional level [5–9]. Interestingly, also high levels of kinesiophobia have been associated with worse outcomes in various diseases affecting the spine [10–12], upper limbs [13, 14] and lower limbs [15, 16]. Kinesiophobia is defined as the fear and refusal of movement [17], based on the fear-avoidance model introduced by Vlaeyen et al. [18]. In patients undergoing knee replacement, authors showed a negative correlation between high levels of kinesiophobia and reduced knee flexion [19, 20], ambulatory performance [21, 22], pain [20, 22] and length of hospital stay [23]. In literature, Kinesiophobia was measured using the Tampa Scale and patients with high levels of Kinesiophobia were identified for scores greater than 40 [21].

Considerable attention has been paid to the role of kinesiophobia with respect to knee prosthesis, however, to our knowledge, it has not yet been studied as a prognostic factor of short-term functional performance following total hip replacement. Therefore, the main aim of the present study is to examine the possible predictors of early functional performance in terms of short-term autonomy of patients undergoing total primary hip arthroplasty, including demographics as age, sex and body mass index, preoperative functional ability, type of anaesthesia, level of haemoglobin, pain before and after surgery and level of kinesiophobia before surgery. Secondly, the study aimed to describe the main characteristics of the population with the highest levels of kinesiophobia and to analyze any differences with the population with lower levels of kinesiophobia.

## Methods

### Study design

Prospective-prognostic cohort study.

### Participants

The present study was carried out at a single-specialized orthopedic hospital in a 33-bed surgical department for hip joint replacement. Patients undergoing primary hip replacement with a minimally invasive and lateral approach were recruited consecutively according to the following inclusion criteria: age range between 18 and 75 years old, symptoms of hip pain at least 3 months before surgery. A period of at least 3 months was deemed necessary for the onset of the mechanisms of fear the of movement, characteristic of kinesiophobia, which present an alteration of function from a clinical point of view [18]. Patients who underwent hip replacement due to trauma or surgical revision, other surgical treatments of lower limbs in the last year, with concomitant rheumatic diseases (rheumatoid arthritis, ankylosing spondylitis) and/or neurological diseases (Parkinson’s disease, stroke) or with signs of cognitive impairment, were excluded from the study. In addition, patients were excluded from the study in cases where orthopedic complications of failed prosthetic implants (dislocations or implant mobilization) or major complications occurring during postoperative hospitalization causing interruptions in the normal rehabilitation process. The study received formal approval from the Institution’s ethics committee and each patient provided written consent. The study was registered on the ClinicalTrials.gov registry (N. NCT02786121).

### Outcome measure

The main outcome is the early level of functional performance achieved by patients measured using the Iowa Level of Assistance (ILOA) scale on the fifth postoperative day. The ILOA scale was studied and validated by Shields et al. [24] and has been used in several studies with populations similar to the present one [6, 25, 26]. The scale presents five items: sitting position, standing position, walking, stair climbing and walking speed. Each activity in the study is measured according to the degree of assistance requested and aid used. Assistance levels were evaluated with scores from 0 to 6, where lower scores indicate greater functional independence. The scale for aids used are evaluated with scores from 0 to 5. The total score ranges from 0 to a maximum of 50. No specific cut-off was settled.

### Study variables

The variables considered and subsequent samples collected were identified before the start of the study through literature research.

*Demographics and clinical variables:* age, gender, body mass index (BMI), type of anesthesia (total, spinal and combination), intensity of pain (Numerical Rating Scale - NRS) [27], hemoglobin variation (calculated as the difference between the preoperative value and the minimum value recorded in the first five postoperative days) and the onset of complications (urinary tract, respiratory tract, cardiovascular system and skin) were the variables of which samples were collected by a clinician and recorded.

*Self-reported measures:* during preoperative admission at the hospital’s orthopedic department, the physiotherapist recruited patients based on inclusion and exclusion criteria, collected study consent forms and administered the following assessment scales:

- the preoperative functional ability was measured by the Italian version of the Western Ontario and McMaster Osteoarthritis Index (WOMAC) [28]. This scale was developed for patients with osteoarthritis of the hip and knee and has been proven to be valid and repeatable [28]. The WOMAC consists of 24 items that are evaluated according to a 5-point Likert scale with scores from 0 to 4 for each item and divided into 3 groups: pain, stiffness and function. Higher scores indicate greater difficulty with an overall score that could range from 0 (no limitation) to 96 (maximum limitation).

- the level of kinesiophobia was measured using the Tampa Scale for Kinesiophobia (TSK). The Italian version [29] provides 13 items each of which uses a Likert 4-point scale that ranges from 1 (strongly disagree) to 4 (strongly agree). The total score is calculated by adding up the scores of each item, the higher scores representing higher levels of kinesiophobia. The total score ranges from 13 (absence of kinesiophobia) to 52 (maximum fear of movement). This variable was collected as a continuous variable for the main purpose of the study in the analysis of predictive factors relating to early functional performance. The evaluation of kinesiophobia was carried out on the preoperative day. In order to meet the second objective of the study and describe the main characteristics of the population with the highest kinesiophobia scores and any differences with the population with lower levels, based on some studies on knee prosthesis [20, 21], a cut-off score of 40 was established. This secondary analysis had the role of providing possible indications for further studies and insights in relation to the role of Kinesiophobia in the processes that lead patients to hip replacement surgery.

### Inpatient physiotherapy program

Physiotherapy during the postoperative phase required two daily 30-min sessions of physical therapy starting from the first postoperative day. The exercise program was carried out early on and accelerated with the aim to achieve upright position on day one, walking with a frame on day two, walking with forearm crutches on day three, and, depending on the patient’s clinical conditions, ascending three steps. Each physiotherapist was free to choose the activities to execute in each session from the physiotherapy schemes. No specific assessment or physiotherapy treatment was planned out regarding patients who had significant anxiety levels. In cases where the physiotherapist considered it appropriate, it was possible to verticalize the patient in two operators to obtain a greater level of safety. The pharmacological treatment set in the postoperative phase did not provide the use of targeted therapy for anxiety, except in cases where treatment was not already being carried out in the patient on a regular basis during the preoperative period.

### Sample size

The ILOA scale in a sample of 167 previously studied patients [6] at the same surgery department had an average score of 16.6 with standard deviation of 6.5 at hospital discharge. The number of patients enrolled in this study was sized on this score and on the number of predictive parameters included in the multivariate statistical analysis. Therefore, based on these considerations, it was estimated to enroll a sample of at least 200 subjects.

### Statistical analysis

All continuous data are expressed in terms of mean ± standard deviation (SD) and categorical variables are expressed as proportions or percentages. The Kolmogorov Smirnov test was performed to test normality of continuous variables. The Spearman’s rank correlation was used to assess correlation between continuous data and the Pearson chi square test, evaluated by the exact method (to manage small subgroups), was performed to investigate the relationships between grouped variables. One-Way ANOVA was performed to assess differences among groups when the Levene test for homogeneity of variances was not significant (*p* < 0.05), alternatively the Mann Whitney test was used. The multivariate analysis was performed by the General Linear Model with the fixed effects as the categorical predictor, and the covariates as the continuous predictor. For all tests, *p <* 0.05 was considered significant. Statistical Analysis was carried out by using the Statistical Package for the Social Sciences (SPSS) software version 15.0 (SPSS Inc., Chicago, USA). In addition to the main analysis described here, a further analysis was conducted to investigate possible differences between the basic characteristics of the sample in relation to high levels of Kinesiophobia, for Tampa Scale scores greater than 40.

## Results

**Fig. 1:**
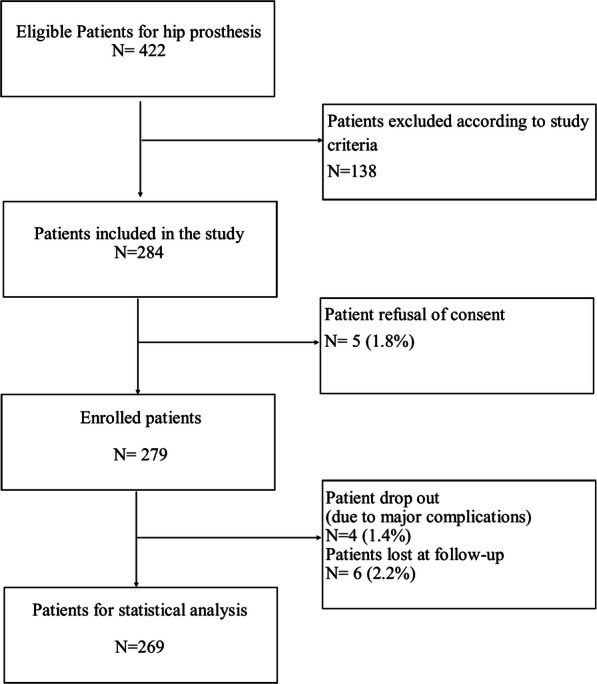
Enrollment process

A total of 284 patients were enrolled between May 2016 and February 2017, according to the inclusion criteria. The flow of patients and the reasons for exclusions are outlined in Fig. 1. Complications occurred in 12 patients (4.5%). Statistical analysis was performed on 269 patients. The description of the sample and of the variables collected and used for statistical analysis is summarized in Table 1 for continuous variables, and in Table 2 for categorized variables. The average ILOA score was 19.5 (DS 8.3).

**Table 1:** Characteristics of the population and univariate analysis between continuous variables and ILOA score

Variables	Mean (DS)	ILOA TOT	
		Rho	***P***value
**Age**	59.9 (10.5)	0.263	< 0.0005
**BMI**	26.9 (4.1)	0.135	0.027
**WOMAC total**	51.9 (14.3)	0.147	0.016
**WOMAC pain**	9.5 (3.4)	0.057	0.351
**WOMAC stiffness**	4.7 (1.7)	0.092	0.134
**WOMAC function**	37.9 (10.1)	0.149	0.015
**Pre TSK**	35.1 (7.8)	−0.360^a^	0.556
**Pre Hemoglobin**	14.0 (1.3)	−0.168	0.006
**Delta Hemoglobin**	4.1 (1.4)	−0.134	0.028

Missing cases: 3 for WOMAC; 6 for pre TSK; 1 for per Hemoglobin and 1 for Delta Hemoglobin

^a^Pearson correlation

**Table 2:** Characteristics of the population and univariate analysis between categorical variables and ILOA score

Variables	Frequency (%)	ILOA Mean (DS)	***P*** value
**Sex**			
Male	118 (43.9)	17.1 (7.5)	< 0.0005
Female	151 (56.1)	21.6 (8.4)	
**Anesthesia**			
Total	26 (9.7)	21.9 (9.6)	0.276
Spinal	13 (4.9)	18.5 (6.7)	
Combination	229 (85.4)	19.4 (8.2)	
**At least one day with pain > 3 (NRS)**			
No	243 (90.7)	19.4 (8.2)	0.458
Yes	25 (9.3)	21.7 (9.0)	

Missing cases: 1 for anesthesia and 1 for pain

The level of kinesiophobia, measured by the Tampa scale, showed an average score of 35.1 (7.8). This variable was not associated with early functional performance measured by ILOA score. Instead, the univariate analysis showed increase in age, increase in body mass index and a more compromised functional preoperative status measured with WOMAC score, as risk factors. Results showed protective factors to be the male sex, a higher preoperative hemoglobin level and a greater reduction in hemoglobin in the postoperative phase.

The significant variables were included in the multivariate analysis model and results are summarized in Table 3. The independent predictive factors include: age, sex and body mass index.

**Table 3:** Multivariate analysis for ILOA

Variables	B	95% Wald confidence interval		***P*** value
		Lower limit	Upper limit	
Age	0.172	0.83	0.261	< 0.001
Male Sex	−4.435	−6.321	−2.55	< 0.001
BMI	0.279	0.048	0.509	0.018

Further analysis was conducted on the characteristics of the sample and postoperative outcomes of patients with a high level of preoperative kinesiophobia. Patients with high preoperative kinesiophobia scores, greater than 40, measured with the Tampa Scale, were 30%. The results of this secondary analysis are shown in Table 4. Patients with higher levels of kinesiophobia before surgery had higher WOMAC score. No further differences emerged between the population with higher and lower levels of kinesiophobia.

**Table 4:** Analysis for Kinesiophobia groups

	Kinesiophobia < 40 ***N*** = 187	Kinesiophobia ≥40 ***N*** = 82	***P***value
*Baseline Characteristic*			
**Age, mean (DS)**	59.5 (10.3)	61.1 (11.0)	0.166
**Female, %**	44.9	41.5	0.689
**BMI, mean (DS)**	27.0 (4.2)	27.2 (4.0)	0.711
**Hemoglobin, mean (DS)**	13.9 (1.3)	14.0 (1.4)	0.592
**Womac pain, mean (DS)**	9.3 (3.3)	10.0 (3.6)	0.079
**Womac stiffness, mean (DS)**	4.6 (1.6)	4.7 (1.9)	0.428
**Womac function mean (DS)**	36.8 (10.1)	39.9 (9.7)	0.011
**Womac total, mean (DS)**	50.7 (13.9)	54.6 (14.2)	0.015
**Pre TSK, mean (DS)**	31.4 (5.6)	44.1 (3.3)	< 0.0005
*Postoperative outcome*			
**Length of hospitalization (LOS) mean (DS)**	5.98 (1.8)	6.04 (1.3)	0.794
**At least one day with pain > 3 (NRS), %**	9.6	8.6	1.000
**ILOA Score, mean (DS)**	20.0 (8.9)	18.8 (6.9)	0.332

## Discussion

In the present study, the increase in age, female sex and the increase in BMI emerged as independent predictors of the worse recovery of patient autonomy after total primary hip replacement; this association did not emerge with respect to kinesiophobia. The recovery of functional autonomy measured by the ILOA scale showed an average score of 19.5 (SD 8.3) on the fifth postoperative day. This value is in line with the average score of 18.2 (DS 7.7) reported by Stockton and Mersegne [26] in which the evaluation was carried out on the sixth postoperative day. Kinesiophobia was highlighted as a significant factor in various studies [19–23, 30], which showed a worse outcome compared to post-operative recovery.

The mean Tampa scale score reported by Doury-Panchout [21] was 36.5 (6.7), slightly higher than the score reported in the present study of 35.1 (7.8) in the preoperative phase. The population with high levels of kinesiophobia had an average score of 43.7 (3.5) and 46.0 (10.2), respectively, in the Doury-Panchout and Guney-Deniz studies, which is in line with the score of the present work of 44.1 (3.3). Moreover, the percentage of patients with a high level of kinesiophobia was 36 in the Doury-Panchout study, and 47.8 in the Guney-Deniz study, while in the present study it was 30. Therefore, in patients who undergo knee replacement, kinesiophobia seems to have the same intensity as patients with osteoarthritis of the hip, but with a higher frequency. This aspect could also explain the difference in the role that this factor plays in the postoperative recovery phase. Fear of movement mechanisms likely to associate with post-operative recovery are probably more common in patients with knee replacement. An association between joint function and kinesiophobia level emerges in patients with hip replacement, who report that fear of movement seems to have a significant role in the process that leads to worsening of function but is not directly associated with preoperative pain symptoms.

It should be borne in mind that the above referenced studies were aimed at patients undergoing knee prosthesis and many of these cases presented an underestimated sample size with possible bias. In Archer et al. [31], in patients undergoing spinal surgery, it was confirmed, instead, that preoperative kinesiophobia was not correlated with functional outcomes evaluated at 6 weeks and 6 months after surgery, while there was an association between the same outcomes and postoperative kinesiophobia. It remains to be clarified what is the role of surgical intervention that modifies the structure of the hip joint and soft tissue in a very short time, with respect to kinesiophobia, which authors have described as a process of pathological adaptation with prolonged modes and timing. Further studies are needed to understand whether a preoperative condition of fear may be significantly correlated with longer-term outcomes. As previously mentioned, increase in age was found to be one of the factors associated with postoperative functional outcome. Indeed, several authors have shown this correlation [5, 6, 32], both with respect to a functional outcome and to the evaluation of the length of hospital stay [33]. In addition, sex emerged as another variable linked to patient characteristics associated with the ILOA score. In fact, women showed a lower level of recovery compared to men, as already highlighted in the studies of Vincent [5], Morri [6] and Elings [34]. In these studies, the age of the female population is slightly more advanced, additionally, their conditions of pain and preoperative function are worse than their male counterparts; these aspects could possibly explain the poorer results of post-operative recovery.

The only factor that emerged as significant, which was possible to intervene during the preoperative phase was BMI. In the literature however, the role of BMI has not yet been defined in a decisive manner. For example, in Vincent’s study [5], BMI did not appear to be significant, while it was significant in the systematic reviews of Buirs [7], Liu [35], Elings [34] and Smith [32].

The preoperative functional ability was not associated with early postoperative recovery. In a literature review by Mak et al. [36], the authors showed how preoperative exercise was prescribed to maintain a better functional level and to reduce pain during the waiting period before surgery, but without associated it with post-operative recovery. On the contrary, Smith’s study [32] and the systematic review of Buirs [7] showed association between preoperative functional ability and postoperative recovery. In addition, a higher hemoglobin level and greater blood loss emerged as protective factors with respect to the functional outcome. Ibrahim et al. [37] showed a correlation between the level of hemoglobin and the length of hospital stay. Therefore, in the present study, it can be hypothesized that hemoglobin played such a confounding role that the multivariate analysis lost significance, confirming the results of Schneider et al. [33].

### Limits

The study presents some limitations. Among the preoperative variables, the comorbidities present were not evaluated. However, this factor does not significantly correlate with functional recovery reported in the literature [34]. Another limit is the length of follow-up. The study was aimed at early functional recovery, while a more prolonged follow-up over time may be useful in a further study to understand the role of kinesiophobia on outcomes in the medium to long term. Finally, the mean ILOA score (16.6 with SD 6.5) from which we started to calculate the sample size of 200 patients, was lower than the mean score that emerged from the study (19.5 with DS 8.3). The studied sample of 269 patients was, however more than originally expected and this allowed us to obtain stable results.

## Conclusion

Based on our findings, early functional performance after hip replacement surgery was not correlated with the level of preoperative kinesiophobia. Three significant factors that describe a population more at risk of not achieving optimal functional performance are older age, female gender, and higher BMI. It is important to take these risk factors into consideration when planning both surgery and rehabilitation approach. In the preoperative phase, patients with high levels of kinesiophobia exhibit more impaired preoperative functional ability.

## Data Availability

The datasets used and/or analysed during the current study are available from the corresponding author on reasonable request.

## Abbreviations

BMI: Body Mass Index
ILOA: Iowa Level of Assistance
NRS: Numerical Rating Scale
WOMAC: Western Ontario and McMaster Osteoarthritis Index (WOMAC)
SPSS: Statistical Package for the Social Sciences
